# Plasticity-Related PKM***ζ*** Signaling in the Insular Cortex Is Involved in the Modulation of Neuropathic Pain after Nerve Injury

**DOI:** 10.1155/2015/601767

**Published:** 2015-09-20

**Authors:** Jeongsoo Han, Minjee Kwon, Myeounghoon Cha, Motomasa Tanioka, Seong-Karp Hong, Sun Joon Bai, Bae Hwan Lee

**Affiliations:** ^1^Department of Physiology, Yonsei University College of Medicine, Seoul 120-752, Republic of Korea; ^2^Brain Korea 21 PLUS Project for Medical Science, Yonsei University College of Medicine, Seoul 120-752, Republic of Korea; ^3^Division of Bio and Health Sciences, Mokwon University, Daejeon 302-729, Republic of Korea; ^4^Department of Anesthesiology and Pain Medicine, Yonsei University College of Medicine, Seoul 120-752, Republic of Korea

## Abstract

The insular cortex (IC) is associated with important functions linked with pain and emotions. According to recent reports, neural plasticity in the brain including the IC can be induced by nerve injury and may contribute to chronic pain. Continuous active kinase, protein kinase M*ζ* (PKM*ζ*), has been known to maintain the long-term potentiation. This study was conducted to determine the role of PKM*ζ* in the IC, which may be involved in the modulation of neuropathic pain. Mechanical allodynia test and immunohistochemistry (IHC) of zif268, an activity-dependent transcription factor required for neuronal plasticity, were performed after nerve injury. After *ζ*-pseudosubstrate inhibitory peptide (ZIP, a selective inhibitor of PKM*ζ*) injection, mechanical allodynia test and immunoblotting of PKM*ζ*, phospho-PKM*ζ* (p-PKM*ζ*), and GluR1 and GluR2 were observed. IHC demonstrated that zif268 expression significantly increased in the IC after nerve injury. Mechanical allodynia was significantly decreased by ZIP microinjection into the IC. The analgesic effect lasted for 12 hours. Moreover, the levels of GluR1, GluR2, and p-PKM*ζ* were decreased after ZIP microinjection. These results suggest that peripheral nerve injury induces neural plasticity related to PKM*ζ* and that ZIP has potential applications for relieving chronic pain.

## 1. Introduction

Nerve injury-induced neural plasticity in the central nervous system (CNS) is one of the important mechanisms involved in chronic pain [1–4]. Long-term potentiation (LTP), being considered as a neural substrate of learning and memory, is an underlying mechanism of synaptic plasticity [5, 6]. Furthermore, several studies have reported that LTP is induced in the spinal cord and the cerebral cortex by acute and chronic pain states [7–9]. Thus, it is believed that there is a common mechanism between learning/memory and chronic pain [10].

Protein kinase M*ζ* (PKM*ζ*) is a key molecule required for the maintenance of late LTP (L-LTP) [11, 12]. It is one of the atypical PKC isoforms, which include PKM*ζ*, PKC*ζ*, PKC*ι*, and PKC*λ*. Because PKM*ζ* has only the catalytic domain of PKC*ζ*, it can be activated persistently [13]. *ζ*-pseudosubstrate inhibitory peptide (ZIP), a selective inhibitor of PKM*ζ*, can reverse LTP maintenance and block L-LTP induction [14, 15]. Furthermore, ZIP can also reverse a variety of memory types, such as spatial information, fear, addiction, and conditioned taste aversion (CTA) memory [15–19]. Several studies have reported that PKM*ζ*-related pain memory can be erased in the spinal cord and the brain [9, 20, 21].

The insular cortex (IC) is one of the pain-processing regions of the brain which is particularly related to the emotional aspects of pain [22–25]. Clinical and animal studies have shown that lesions of the IC cause pain asymbolia and can reverse the neuropathic pain state [26–28]. NMDA receptor-dependent plastic changes after nerve injury and PKM*ζ*-mediated CTA memory in the IC have been reported [16, 29]. However, there have been no studies published on immediate-early gene (IEG) expression with respect to LTP and PKM*ζ*-related mechanisms of plasticity in the IC after nerve injury.

Zif268, which is also known as early growth response protein 1 (EGR-1), is required for consolidation of L-LTP in the hippocampus and the spinal cord [30, 31]. In order to determine whether there is a possibility of LTP induction in the IC after nerve injury, an immunohistochemical study on zif268 was conducted. Moreover, in order to reveal whether PKM*ζ* signaling in the IC is involved in the maintenance of neuropathic pain, expression levels of PKM*ζ* and phospho-PKM*ζ* (p-PKM*ζ*) were measured after ZIP injection into the IC, and analgesic effects following ZIP injection into the IC were observed.

## 2. Materials and Methods

### 2.1. Experimental Animals

Experimental protocols in this study complied with the National Institute of Health guidelines and were approved by the Institutional Animal Care and Use Committee of the Yonsei University Health System. Adult male Sprague-Dawley rats (Koatec, Pyeongtaek, Korea, 250–280 g) were used. Rats were allowed to accommodate to the colony room for 7 days after arrival and were housed in plastic cages. Rats were maintained under a 12-hour light/dark cycle with food pellets and water provided* ad libitum*.

### 2.2. Cannulation and Neuropathic Surgery

For cannula implantation, rats were anesthetized with sodium pentobarbital (50 mg/kg, i.p). 28-gauge guide cannulas were bilaterally implanted into the IC (AP: +1.0 mm, ML: ±4.7 mm, and DV: −5.8 mm) on a stereotaxic frame. Rats were allowed to recover for 7 days after cannula implantation. Neuropathic pain surgery was performed after recovery. Cannula-implanted rats were anesthetized with sodium pentobarbital (50 mg/kg, i.p.) and branches of the left sciatic nerve were exposed. The tibial and sural nerves were tightly ligated with 4-0 black silk and cut, while the common peroneal nerve was left intact [32]. The same procedure was applied to the sham-operated group without nerve injury. The wound was sutured in the muscle and skin layers.

### 2.3. Behavioral Test for Mechanical Allodynia

In order to observe the development of neuropathic pain, the mechanical allodynia test was performed by an experimenter blinded to the experiment before nerve injury, on postoperative days (PODs) 1 and 3. Rats were placed on a metal mesh floor under rectangular-shaped transparent domes. Animals were familiarized to the test conditions for 15 minutes before testing began. Withdrawal threshold was assessed by the application of electric von Frey filament (UGO Basile, Varese, Italy) stimulation to the sensitive area of the nerve-injured hind paw. This measurement was conducted 8 times at intervals of 2-3 minutes. The mechanical force was recorded when withdrawal responses of the hind paw occurred. Positive responses to mechanical stimulation included licking, sudden withdrawal, and biting of the ipsilateral paw.

### 2.4. ZIP Microinjection into the Insular Cortex and Analgesia Test

ZIP microinjection was conducted on POD 3. A Hamilton syringe and PE-10 tubing were used with an injection cannula for microinjection. Saline (0.9% NaCl) or 10 nmol/*μ*L of ZIP (Tocris Bioscience, Minneapolis, MN, USA) diluted in saline was infused into the IC bilaterally, 0.5 *μ*L per side. After injection, the injection cannulas were maintained in position as they were for at least 1 minute. The behavioral test was performed before ZIP injection and 30 minutes and 1, 2, 4, 8, 12, 24, and 48 hours after microinjection.

### 2.5. Immunofluorescence Double Staining

For double immunofluorescence staining, rats were deeply anesthetized with urethane and perfused transcardially with saline followed by 4% paraformaldehyde in 0.1 M sodium phosphate buffer (PB, pH 6.8). The brain was removed from the skull and postfixed with 4% paraformaldehyde in PB at 4°C overnight. After postfixation, the brain block was transferred into phosphate-buffered saline (PBS, pH 7.4) containing 30% sucrose for 24 hours. The brain sample was covered with a cryosection compound (frozen section compound FSC 22, Leica, Wetzlar, Germany) and was frozen in −70°C deep freezer. Sample tissues were cut on a coronal section of 30 *μ*m thickness on a cryostat (HM 525, Thermo Scientific, Waltham, MA, USA). Section slides were then washed 3 times with 1x Tris-buffered saline (TBS) containing 0.025% Triton X-100. After washing, the section slides were incubated in 10% normal goat serum (Vector Laboratories, Burlingame, CA, USA) with 1% bovine serum albumin (BSA, Thermo Scientific) in TBS for 1 hour at room temperature. The sections were incubated overnight in rabbit monoclonal anti-zif268 antibody (1 : 200, Santa Cruz Biotechnology, Santa Cruz, CA, USA) and mouse monoclonal anti-NeuN antibody (1 : 200, Abcam, Cambridge, UK) diluted in 10% normal goat serum with TBS containing 1% BSA at 4°C. The sections were then rinsed 2 times with 1x TBS plus 1% Tween-20. After washing 2 times for 10 minutes, sections were incubated in secondary antibodies which were goat anti-rabbit Alexa Fluor 488 (1 : 200, Abcam) and goat anti-mouse Alexa Fluor 647 (1 : 200, Abcam), diluted in 10% normal goat serum with TBS containing 1% BSA for 1 hour at room temperature. Finally, the slides were washed 2 times with 1x TBS containing 0.025% Triton X-100 and mounted in Vectashield mounting media (Vector Laboratories).

### 2.6. Immunohistochemistry

To calculate the population of zif268-positive cells, nickel-enhanced 3,3′-diaminobenzidine (DAB) immunostaining was performed on POD 3. Briefly, rat brain slices were prepared using the same steps as for immunofluorescence staining. For zif268 staining, section slides were rinsed 5 times with 1x PBS and incubated in methanol with 0.3% H_2_O_2_ for 15 minutes to inhibit endogenous peroxidase activity. After washing the section slides 5 times, the sections were incubated with PBS containing 1% normal horse serum (Vector Laboratories) for 30 minutes and then incubated overnight at 4°C in 0.3% Triton X-100, 2% normal horse serum (Vector Laboratories), and rabbit monoclonal anti-zif268 antibody (1 : 4,000, Santa Cruz Biotechnology). The sections were rinsed 5 times with 1x PBS and incubated for 30 minutes in a universal biotinylated anti-mouse/rabbit secondary antibody (1 : 50, Vector Laboratories). Section slides were then washed again and incubated for 30 minutes with PBS containing avidin-biotinylated horseradish peroxidase complex (1 : 50, Vector Laboratories). Following washing 5 times for 15 minutes, sections were incubated for 5 minutes in a solution containing 0.1% of DAB and 0.1% ammonium nickel sulfate in 1x PBS and 0.01% H_2_O_2_. Finally, the sections were washed to stop the DAB reaction, serially dehydrated in 50, 70, 95, and 100% ethanol, cleared in xylene, and coverslipped with Permount (Fisher Scientific, Waltham, MA, USA).

To quantify zif268-positive cells in the IC, 8 representative sections of the IC were chosen from each brain. The interval of each section was 300 *μ*m. Therefore, 8 sections cover anteroposterior range (2.1 mm) of the IC. Zif268-positive cells in the IC were identified with reference to the brain atlas of Paxinos and Watson [33]. All of the zif268-labeled cells from light-field microscopy image (×20 objective, Olympus BX40, Olympus, Tokyo, Japan) were counted manually. An experimenter blinded to the treatment conditions counted zif268-labeled cells of the ipsilateral and contralateral sides.

### 2.7. Western Blotting

To collect insular cortices, animals were anesthetized with enflurane and decapitated. The ipsilateral and contralateral rostral insular cortices were quickly isolated and transferred into a deep freezer. Extracted samples were stored at −70°C. For protein extraction, samples were homogenized by sonication in lysis buffer (Proprep, iNtRON Biotechnology Inc., Seongnam, Korea) containing phosphatase inhibitor (PhosStop, Roche, Penzberg, Germany). Samples were centrifuged at 22,250 g for 10 minutes at 4°C and supernatants were collected, and total protein concentrations of lysates were assessed with a spectrophotometer (ND-1000, NanoDrop Technologies Inc., Wilmington, DE, USA). 10 *μ*L of protein of brain tissue extracts was denatured per well and run on 10% Bis-Tris gels (Bio-Rad, Hercules, CA, USA) for detection of PKM*ζ*, p-PKM*ζ*, GluR1, and GluR2. Proteins were transferred onto polyvinylidene difluoride (PVDF) membranes (GE Healthcare, Buckinghamshire, UK). Membranes were blocked in 5% skim milk in TBS with Tween-20 for 1 hour and incubated in primary antibodies overnight on a rocking platform at 4°C. Primary antibodies against PKM*ζ* (1 : 2,000, Cell Signaling Technology, Beverly, MA, USA), p-PKM*ζ* (1 : 2,000, Cell Signaling Technology), GluR1 (1 : 2,000, Millipore, Temecula, MA, USA), GluR2 (1 : 2,000, Abcam), and GAPDH (1 : 10,000, Ab Frontier, Seoul, Korea), which was used as a loading control, were used for western blotting. On the following day, the membranes were incubated in the appropriate secondary antibodies for 2 hours and horseradish peroxidase activity was visualized using a chemiluminescent substrate (ECL Prime western blotting detection reagent, GE Healthcare) and processed with a local allocation system (LAS) (ImageQuant LAS 4000 Mini, GE Healthcare). The intensity of the bands for PKM*ζ*, GluR1, and GluR2 was normalized to the intensity of GAPDH. The intensity of the bands for p-PKM*ζ* was normalized to the intensity of PKM*ζ*.

### 2.8. Statistical Analysis

Unpaired *t*-test for post hoc comparison was used for two-group comparisons while two-way ANOVA with repeated measures was used to analyze behavioral test. Western blots and IHC were compared using an unpaired *t*-test. All data were expressed as the mean ± SEM. A *P* value less than 0.05 was considered statistically significant.

## 3. Results

### 3.1. Development of Neuropathic Pain

**Figure 1:**
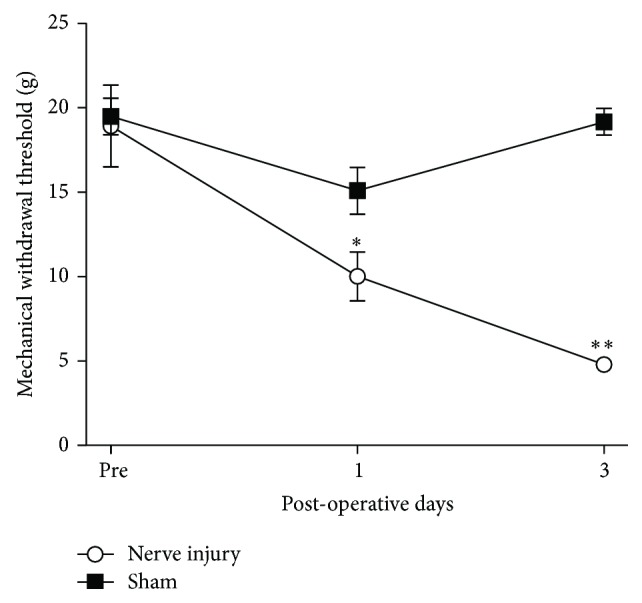
Development of mechanical allodynia after nerve injury. On PODs 1 and 3, rats developed significant neuropathic pain compared to sham group (^∗∗^ *P* < 0.01).

Injury to two major branches (sural and tibial nerves) of the sciatic nerve induced mechanical allodynia on PODs 1 and 3 (Figure 1). Repeated measures two-way ANOVA indicated effects of group (*F*_1,13_ = 37.520, *P* < 0.001), PODs (*F*_2,26_ = 13.613, *P* < 0.001), and interaction between group and PODs (*F*_2,26_ = 10.305, *P* < 0.01). The mechanical threshold of nerve-injured group decreased on POD 1 (*n* = 8, *P* < 0.01, unpaired *t*-test) and POD 3 (*n* = 8, *P* < 0.01, unpaired *t*-test) relative to sham group (*n* = 8).

### 3.2. Immunofluorescence Double Labeling of Zif268 and NeuN

**Figure 2:**
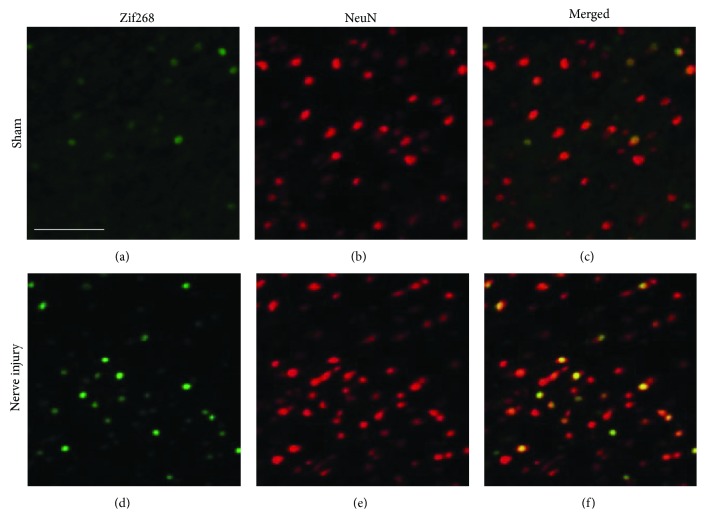
Fluorescence images of zif268 expression in the IC of nerve-injured and sham groups. (a) The sham group showed little expression of zif268-positive cells, unlike the nerve-injured group. (b) NeuN, a neuronal marker (red), was expressed in the sham group. (c) Colocalization of zif268 (green) and NeuN (red) is observed in the sham group. (d) In the nerve-injured group, the distribution of zif268 expression (green) was denser than in the sham group. (e) As in (b), NeuN was expressed in the nerve-injured group. (f) As in (c), colocalization of zif268 (green) and NeuN (red) is observed in the nerve-injured group. Scale bar, 50 *μ*m.

To confirm that zif268 was co-labeled with NeuN in the IC, double labeling of zif268 and NeuN was performed. The representative images of the nerve-injured group are shown in Figures 2(d), 2(e), and 2(f), and those of the sham group are shown in Figures 2(a), 2(b), and 2(c). Zif268 immunoreactivity (green) was observed in the IC (Figures 2(a) and 2(d)). NeuN, a neuronal marker (red), was observed in the IC (Figures 2(b) and 2(e)). Colocalization of zif268 (green) and NeuN (red) was detected in the IC (Figures 2(c) and 2(f)). As shown in Figure 2, zif268-positive cells were colocalized with NeuN-positive cells. This result indicates that zif268 is expressed in IC neurons. Nerve-injured rats have more zif268-positive cells (Figure 2(d)) than the sham group rats (Figure 2(a)). The number of NeuN-positive cells in the IC is similar between the nerve-injured (Figure 2(e)) and sham-operated groups (Figure 2(b)). The merged data of zif268 and NeuN expression show that the IC has a relationship with neuropathic pain (Figures 2(c) and 2(f)).

### 3.3. Immunohistochemistry of Zif268 in the Insular Cortex

**Figure 3:**
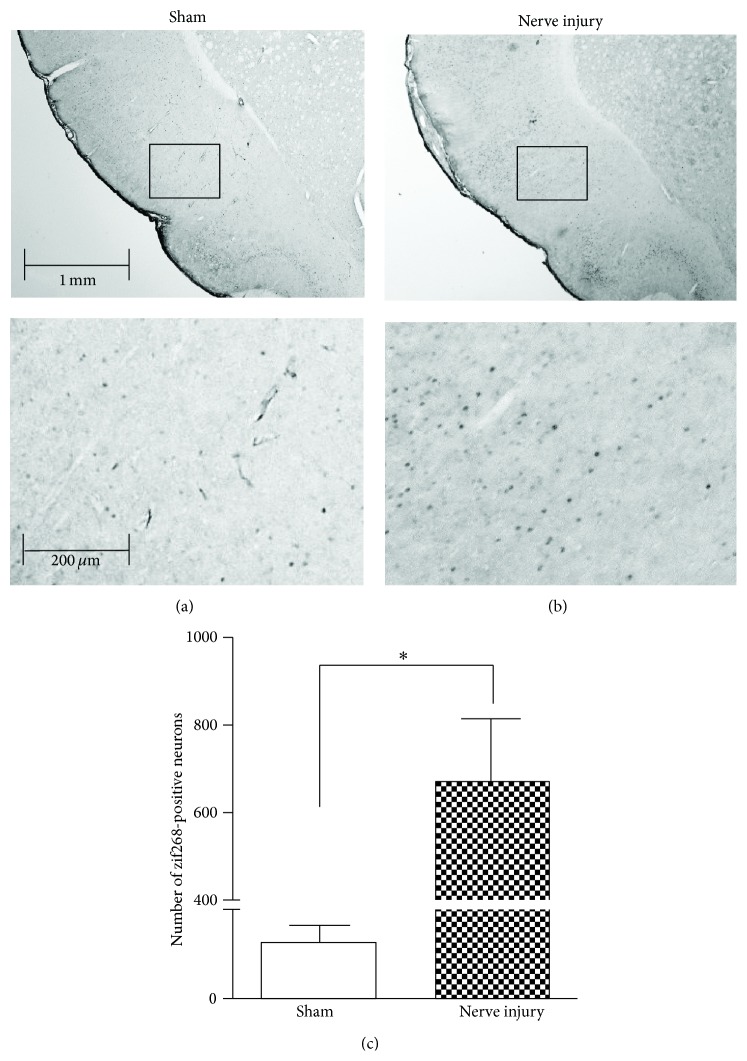
Zif268-positive cells in the IC. (a) Zif268-positive cells in the IC of a sham-operated rat. (b) Zif268-positive cells in the IC of a nerve-injured rat. (c) Comparison of zif268-positive cells in the nerve-injured and sham groups. Zif268-positive cells increased significantly in the IC after nerve injury (^∗^ *P* < 0.05). Cell counts are expressed per section.

Zif268-positive cells were found in the IC of nerve-injured rats (Figures 3(a) and 3(b)). Furthermore, zif268 immunohistochemistry was performed to quantify the zif268-positive cells in the nerve-injured and sham groups. The results showed that the number of zif268-positive cells in the nerve-injured group was significantly increased compared to that of the sham group (*n* = 6, *P* < 0.05, unpaired *t*-test; Figure 3(c)).

### 3.4. ZIP Injection into the Insular Cortex

**Figure 4:**
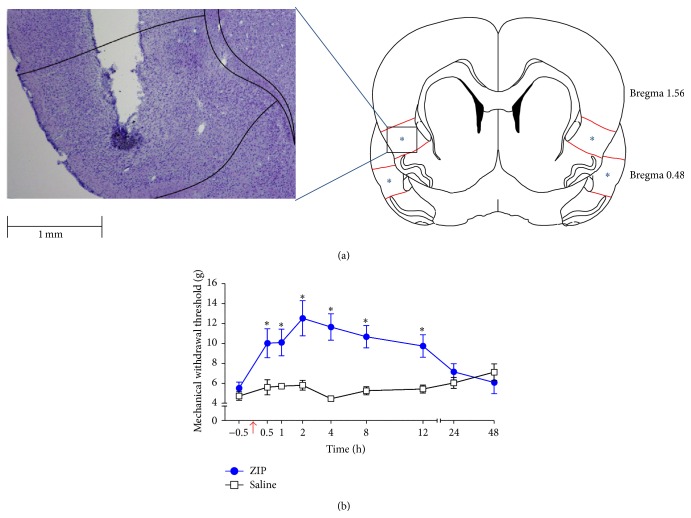
Reduction of mechanical allodynia after ZIP microinjection into the IC. (a) Identification of the ZIP injection site. ZIP was microinjected into the IC. (b) Paw withdrawal threshold to mechanical stimulation in POD 3 rats after microinjection of ZIP. Significant differences between nerve-injured and sham groups were found at the time points from 30 minutes to 12 hours after injection (^∗^ *P* < 0.05). The arrow indicates the time point of ZIP or saline injection.

Figure 4(a) shows the injection site of the IC. Injection of ZIP into the IC decreased mechanical allodynia gradually on POD 3. Repeated measures two-way ANOVA indicated effects of group (*F*_1,9_ = 11.798, *P* < 0.01), time (*F*_7,63_ = 4.23, *P* < 0.01), and interaction between group and time (*F*_7,63_ = 3.93, *P* < 0.01). The time course of mechanical allodynia in the ZIP-injected group (*n* = 7) on POD 3 shows that the analgesic effects of ZIP last for 12 hours after injection (*P* < 0.05, unpaired *t*-test; Figure 4(b)), where analgesia was measured relative to the saline-injected group (*n* = 5). However, at 24 and 48 hours after injection, ZIP had no significant effect (*P* > 0.05, unpaired *t*-test).

### 3.5. Effects on PKM*ζ* and p-PKM*ζ* Expression of ZIP Microinjection into the Insular Cortex

**Figure 5:**
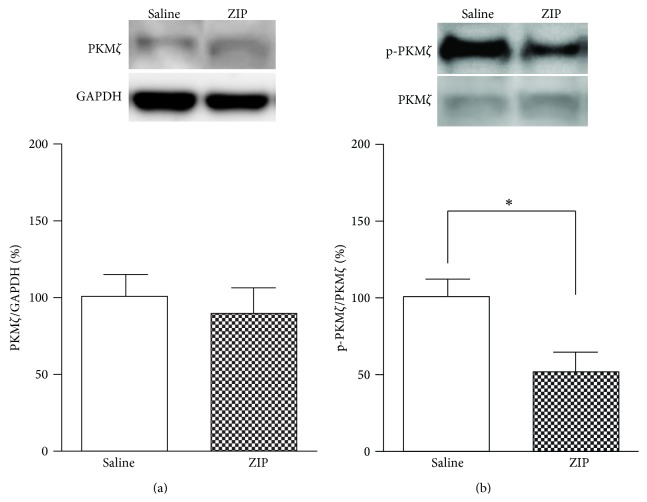
Expression levels of PKM*ζ* and p-PKM*ζ* after ZIP microinjection into the IC on POD 3. (a) Expression levels of PKM*ζ* normalized to GAPDH levels. There was no significant difference in PKM*ζ* levels between the saline injection and ZIP injection groups (*P* > 0.05). (b) Expression levels of p-PKM*ζ* normalized to PKM*ζ* levels. After ZIP injection, p-PKM*ζ* levels were significantly decreased (^∗^ *P* < 0.05).

To determine the role of ZIP, the IC was punched 3 hours after ZIP injection. The total PKM*ζ* in the ZIP-injected group (*n* = 6) was not changed (*P* > 0.05, unpaired *t*-test; Figure 5(a)) on POD 3 relative to the saline-injected group (*n* = 6). However, the expression level of p-PKM*ζ* was downregulated by ZIP injection into the IC on POD 3 (*P* < 0.05, unpaired *t*-test; Figure 5(b)).

### 3.6. Effects of ZIP Microinjection into the Insular Cortex on GluR1 and GluR2 Levels

**Figure 6:**
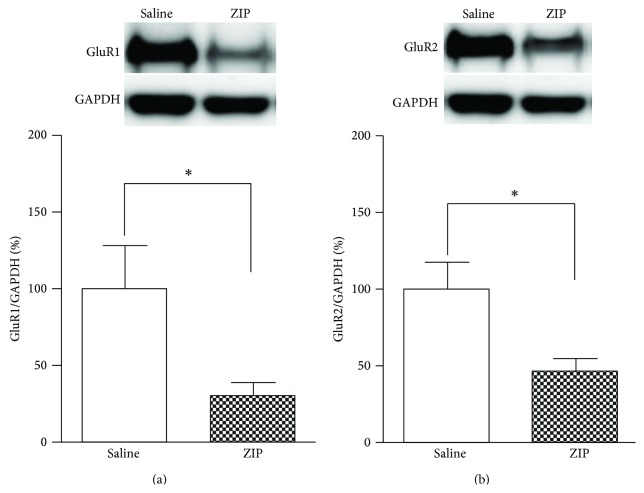
Expression levels of GluR1 and GluR2 after ZIP microinjection into the IC on POD 3. (a) Expression levels of GluR1 normalized to GAPDH levels. There was a significant difference between GluR1 levels in the saline injection and ZIP injection groups. After microinjection of ZIP, levels of GluR1 were significantly decreased (^∗^ *P* < 0.05). (b) Expression levels of GluR2 normalized to GAPDH levels. After ZIP injection, GluR2 levels were significantly decreased (^∗^ *P* < 0.05).

We speculated that the effects of ZIP may contribute to inhibition of AMPA receptors. Accordingly, the expression levels of the AMPA receptor subunits GluR1 and GluR2 were measured after ZIP microinjection on POD 3. The results showed decreased GluR1 and GluR2 levels (*P* < 0.05, unpaired *t*-test; Figures 6(a) and 6(b), resp.) in the ZIP-injected group (*n* = 8), relative to the saline-injected group (*n* = 8).

## 4. Discussion

The IC plays a role in interpretation of emotional aspects of pain as one of the limbic system areas [22]. Several studies have reported that plastic changes in the IC are induced after peripheral nerve injury [29, 34]. Therefore, we focused on plastic changes of the IC and its pain modulation after nerve injury in order to understand the mechanisms of chronic pain. Recent reports have shown that functional changes in the brain following nerve injury may be mediated by LTP [2, 9]. PKM*ζ* plays a key role in maintaining LTP, and inhibition of PKM*ζ* can reverse the chronic pain state and can erase established memories [12, 18]. Accordingly, the present study was performed to determine the plastic changes related to LTP formation, maintenance after nerve injury, and the role of the IC in pain modulation. Our findings suggest that PKM*ζ* in the IC can lead to nerve injury-induced plasticity which contributes to the maintenance of neuropathic pain states.

Zif268 is a well-known marker related to LTP and neuronal plasticity [30]. In the hippocampus, the L-LTP phase requires the expression of IEGs such as zif268 and activity-regulated cytoskeletal-associated protein (Arc) [35, 36]. LTP-inducing stimulation increases zif268 expression levels in the hippocampus [37]. Furthermore, zif268 knockout mice displayed an absence of L-LTP and impaired long-term memory [30]. c-Fos is a neuronal activation marker and is expressed highly after nerve injury in the spinal cord and anterior cingulate cortex (ACC) [9]. Expression of c-Fos in the IC is increased by stress-induced hyperalgesia [38]. However, since zif268 is more correlated with LTP and plasticity to a greater degree than is c-Fos [39], changes in zif268 expression level in the IC after nerve injury were assessed immunohistochemically in our study. There have been several reports that zif268 is induced by nociceptive stimulation [39, 40]. However, there have been no reports on zif268 expression related to nerve injury-induced plasticity in the IC. Our results show that the number of zif268-positive cells increased after nerve injury, which is related to nerve injury-induced LTP in the IC.

Animals with lesions of the IC show reversed neuropathic or inflammatory pain behavior [27, 28]. Patients with lesions in the IC did not display appropriate responses to pain, but all other sensory and cognitive functions still remained intact [26]. Moreover, painful stimuli activate the IC, and direct stimulation of the IC can evoke sensations of pain, indicating that the IC is pronociceptive and involved in the interpretation of pain sensation [23, 41, 42].

PKM*ζ* is a key molecule for maintaining L-LTP and inhibition of PKM*ζ* can erase established LTP [12, 18]. Although the functions of PKM*ζ* were reported to be controversial in some studies [43–45], recent studies have suggested that atypical PKCs compensate the PKM*ζ* role of maintaining LTP in the constitutive PKM*ζ* knockout mice [46]. In fact, many studies have reported that the administration of ZIP into the spinal cord can reverse inflammatory pain, but not neuropathic pain [20, 21, 47, 48]. In contrast, ZIP injection into the ACC reverses the neuropathic pain state, but the analgesic effects disappear 24 hours after injection of ZIP [9]. Based on this report, the effects of ZIP injection into the IC were investigated in the present study. The effects of ZIP lasted for 12 hours after injection into the IC. Our findings suggest that nerve injury induces plasticity related to PKM*ζ* in the IC and that the IC has a pain modulation function. Interestingly, the pain-relieving effect was not permanent. This phenomenon has been observed in other reports utilizing neuropathic pain models. This may be due to the reestablishment of LTP by the tonic peripheral afferent drive [9, 48].

PKM*ζ* is selectively upregulated in the spinal cord by formalin, capsaicin, and nerve injury [21, 48]. In the ACC, both PKM*ζ* and p-PKM*ζ* increased after nerve injury [9]. PKM*ζ* is activated by phosphorylation and can be inhibited by ZIP [18]. Increased levels of p-PKM*ζ* in the ACC contribute to neuropathic pain [9]. Similarly, increased levels of p-PKM*ζ* in the spinal cord contribute to formalin-induced pain [47]. Taken together, we hypothesized that upregulation of p-PKM*ζ* might contribute to neuropathic pain states and that ZIP can alleviate neuropathic pain. Indeed, decreased levels of p-PKM*ζ* were observed following ZIP injection. However, the expression levels of PKM*ζ* did not change after ZIP injection. In contrast, intrathecal infusion of ZIP did not reduce p-PKM*ζ* levels in the spinal cords of formalin model rats [47]. Although our findings are inconsistent with these studies, our results show that the function of p-PKM*ζ* in the IC differs from that in the spinal cord, and these results are in line with those found with respect to the ACC [9].

Several reports have suggested that trafficking of the GluR2 subunit of the AMPA receptor in the hippocampus is related to PKM*ζ* [12, 49]. In addition, fear memory is maintained by PKM*ζ*-mediated GluR2-dependent AMPA receptor trafficking in the amygdala [50]. Other reports suggest that the GluR1 subunit of the AMPA receptor is involved in the molecular machinery of cocaine-induced plasticity in the nucleus accumbens (NAc) [51, 52]. PKM*ζ*-mediated LTP expression may be induced by N-ethylmaleimide-sensitive factor/GluR2-dependent trafficking in the hippocampus [49]. Interestingly, our results show that both GluR1 and GluR2 levels are downregulated by ZIP injection in the neuropathic pain model rats. Consistent with this, GluR1 levels were decreased after ZIP injection into the ACC of neuropathic pain model [9]. Other reports suggest that activation of metabotropic GluR1 (mGluR1) is required for insular L-LTP induction [53]. In addition, administration of ZIP into the NAc core can abolish long-term drug reward memory by effect on GluR2-containing AMPA receptors [54]. Taken together, we assume that PKM*ζ* maintains pain-related long-term plastic changes in the IC via both the GluR1 and GluR2 subunits of AMPA receptors.

## 5. Conclusion

In this study, we demonstrated that there is a correlation between PKM*ζ* and neural plasticity of the IC, which might explain chronic pain mechanisms. PKM*ζ* seems to mediate this plasticity by regulating AMPA receptors containing GluR1 and GluR2. Moreover, the pain modulation function of the IC related to PKM*ζ* was revealed through administration of ZIP. Pharmacological targeting to inhibit plasticity of the IC may therefore present a strategy for chronic neuropathic pain therapy.
